# Spatio-Temporal Interpolation Is Accomplished by Binocular Form and Motion Mechanisms

**DOI:** 10.1371/journal.pone.0000264

**Published:** 2007-02-28

**Authors:** Farid I. Kandil, Markus Lappe

**Affiliations:** Department of General Psychology, Westfälische Wilhelms University of Münster, Münster, Germany; University of Minnesota, United States of America

## Abstract

Spatio-temporal interpolation describes the ability of the visual system to perceive shapes as whole figures (Gestalts), even if they are moving behind narrow apertures, so that only thin slices of them meet the eye at any given point in time. The interpolation process requires registration of the form slices, as well as perception of the shape's global motion, in order to reassemble the slices in the correct order. The commonly proposed mechanism is a spatio-temporal motion detector with a receptive field, for which spatial distance and temporal delays are interchangeable, and which has generally been regarded as monocular. Here we investigate separately the nature of the motion and the form detection involved in spatio-temporal interpolation, using dichoptic masking and interocular presentation tasks. The results clearly demonstrate that the associated mechanisms for both motion and form are binocular rather than monocular. Hence, we question the traditional view according to which spatio-temporal interpolation is achieved by monocular first-order motion-energy detectors in favour of models featuring binocular motion and form detection.

## Introduction

Spatio-temporal interpolation is one of those integrative components underlying our visual experience so perfectly that one would hardly ever think that there is a problem at all. When a car is parked behind a picket fence one can see only a number of narrow stripes of it through the slits. However, when the car starts moving, the percept changes drastically. Rather than seeing a series of successive narrow views of the car, all emerging spatially from the same slits in the fence, what we perceive is a car interpolated in space and time, i.e. a constantly visible unsegregated whole object [e.g. refs. 1]–[5].

Two rival mechanisms, ‘retinal painting’ and ‘spatio-temporal receptive fields’, have been proposed to explain how the visual system recombines the incoming slit-views into a complete image again. Retinal painting theory states that the eyes follow the (global) motion of the car thereby placing incoming slit-views of the car next to one another in the original order on the retina. In contrast, spatio-temporal receptive field theory assumes the existence of receptive fields oriented in space-time. For these receptive fields, space and time are to a certain extent interchangeable, allowing objects that appear *delayed in time* to be considered *displaced in space*. The receptive fields would then place the incoming slit-views into the correct order by means of internal computations rather than external eye movements. Despite their great differences, both theories rely on the correct detection of both form (the series of views) and motion direction in order to link neighbouring views in the right order. Here we investigate whether the underlying mechanism is monocular or binocular by probing the motion and form information it can use.

Throughout the history of spatio-temporal interpolation displays, various kinds of stimulus configurations have been used. In the first series of experiments, objects like letters, geometric figures and animals crossed a single but wide slit or ‘aperture’ [e.g. refs. 1], [ 4]–[6]. In these experiments, both motion direction and local form information emerges from within the one single slit. In the second kind of display, large figures are visible through a number of equally spaced narrow slits [7], [8] allowing subjects to perceive the global contour of the form in most single frames of the presentation. Although each slit is too narrow to detect the motion direction *within* it, subjects readily perceive the motion direction of the global form that they have already detected.

The third kind of display presents multi-slit views in combination with objects that are smaller than the distance between two slits [e.g. refs. 2], [3], [8], [9], [see also refs. 10], [11]. Here, objects are visible through only a single slit at any given point in time, while the global motion direction can be deduced only from the course the objects take between slits. This disentanglement of form and motion cues allowed us to investigate the nature of the associated form and motion detection separately. For each cue, form and motion, we tested whether the interpolation process uses either (i) only monocular, (ii) only binocular or (iii) monocular and binocular information. We used dichoptic masking and interocular completion stimuli to differentiate between these alternatives.

In the critical conditions of the *Dichoptic Masking* experiments (1A, 1B & 2A), stimuli are presented dichoptically to the two eyes. Each monocular view for itself contains valid and sufficient information. However, the two monocular views are constructed in such a way that they mask each other when fused into a binocular view and provide thus only ambivalent information to any purely binocular processor.

In the critical conditions of the *Interocular Completion* experiments (1C & 2B), stimuli are also presented dichoptically. However, there monocular views by themselves do not carry any valid information, thereby excluding monocular processes from the interpolation. Rather, the valid information can only be obtained by binocular interpolation mechanisms, that is after the monocular views have been summed across eyes into a binocular view.

The results obtained here clearly show that only binocular form and motion mechanisms are involved, whereas monocular information does not provide any significant contribution as to the interpolation process. This questions the previously mentioned hypothesis according to which interpolation is computed by a unitary monocular spatio-temporal mechanism [3], [8].

## Methods

### Standard Stimulus

**Figure 1:**
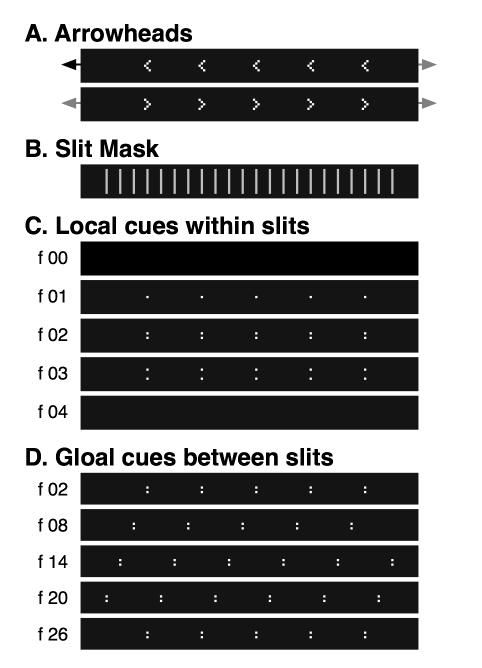
Layout of the standard stimulus. The standard stimulus consists of a number of regularly arranged arrowheads (A), all of which pointed either to the left or to the right. This band was shifted dot-wise either to the left or to the right behind a mask (B), which in turn comprised regularly spread slits of 1 dot width. Distances between arrowheads in the stimulus band were integer multiples of the distance between slits; so same information was visible simultaneously within various slits. (C) During the crossing of one slit, only a small fraction of the arrowheads is physically visible at each point in time. Depending on the arrowhead orientation as well as on the motion direction, the two dots visible within the slits either diverged (as shown here) or converged. (D) Across multiple slit crossings the global motion direction emerged. Both, global cues between slits and local cues within slits must be registered in order to obtain the correct response, i.e. the original arrowhead orientation.

The layout of the standard stimulus is depicted in Figure 1. The stimulus (Fig. 1A) consists of a number of arrowheads, all equal in size (3 dots wide and 5 high) and all pointing in the same direction (left or right). It is moved dot-by-dot behind a slit mask (Fig. 1B) with slits regularly spread every twelve dots. These slits are only one dot wide, hence allowing only one (vertical) pair of dots of the arrowheads to be seen within one slit at any given point in time. Further, arrowheads are equidistant from one another with the midpoint-to-midpoint distance being integer multiples of the space between two slits, i.e. the ‘inter-slit distance’. Thus, the amount of information does not increase with the number of arrowheads visible at the same time. Presentation of more than one arrowhead was necessary in order to facilitate dichoptic masking and interocular completion paradigms.

The first five frames (frames 00 to 04) of an example sequence are shown in Figure 1C. A stimulus band with arrows pointing to the left is moved to the left behind the slit mask (cf. the example indicated in Fig. 1A). Before the arrows pass their first slit, the screen is completely dark (f 00 in Fig. 1C). In the next frame (f 01), only the first dot of each arrowhead is visible. Then the upper and the lower lines of the arrowheads appear by one dot each, with the dots moving further apart from one another (f 02 and f 03). After passing their first and before entering their second slit, the arrows are completely hidden by the mask and the screen becomes entirely dark again (frames 04 to 06). With subsequent frames, the arrows reach and pass subsequent slits (Fig. 1D).

### Ambiguity

Each of the four alternatives shown in Figure 1A produces different motion and form cues both inside and between the slits. The global motion direction *between the slits* solely depends on the motion direction of the arrowheads. In contrast, the local cues, that is, whether the dots diverge or converge *within the slits* is determined by the combination of the motion direction and orientation of the arrowheads. Hence, in order for any mechanism to inversely obtain the original arrowhead orientation always both, local and global cues must be registered.

### Strategy and subject's task

Each of the four experiments comprised four conditions, a critical dichoptic condition (‘dich’), a left and a right monocular control (‘monoL’ and ‘monoR’) and a binocular control (‘bino’). According to the requirements of the experiment, either only the monocular or only the binocular views of the dichoptic stimulus were reliable. In the monocular and binocular conditions, monocular or else binocular views of these stimuli were presented as controls.

In each experiment, twenty trials were presented per condition in a randomly interleaved manner. After each trial, subjects had to indicate the pointing direction of the arrowheads in a two-alternative forced-choice (2-AFC) task by pressing the according cursor key on a standard computer keyboard. They were told to guess the correct answer whenever they could not perceive single arrowheads.

A threshold of 75% (midway between perfect and chance level) distinguished between performance on chance and significant level. This is slightly more conservative than the adjusted threshold derived from the binomial distribution (70.9%).

Subjects were free to move their eyes. Eye movements were not recorded.

### Means of presenting monocular and dichoptic stimuli

**Figure 2:**
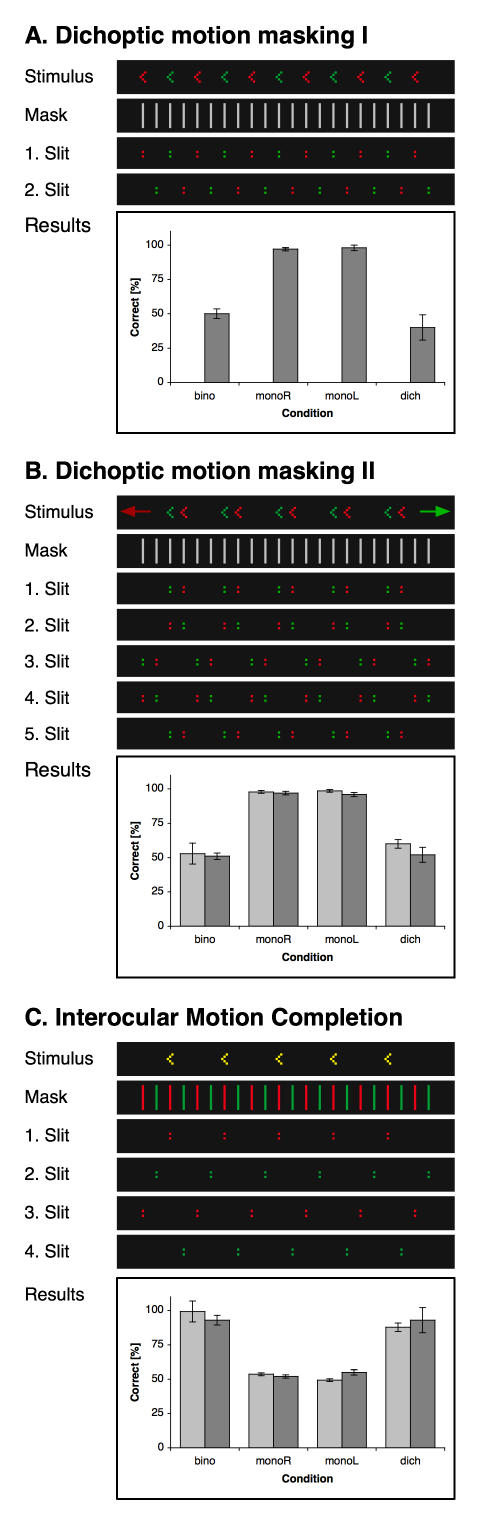
Motion detection. (A) In the critical dichoptic condition, two monocular sets of arrowheads with the same arrowhead orientation and motion direction are superimposed with a phase difference of 180 deg. Thus, only monocular but not binocular detectors are presented with unambiguous motion information. Results show clearly that spatiotemporal interpolation mechanisms have no access to monocular motion. Note that in this experiment and in 1B, all stimuli were always shown as red dots on dark ground and that colour coding is introduced here for the sake of clarity: Red, green and yellow mark stimuli presented to the right or left eye or by both eyes, respectively. (B) Two monocular sets of arrowhead stimuli are superimposed in the critical condition of this task. In both, arrowheads point to the same side but move into opposite directions. As a result, only interpolation mechanisms reading out monocular motion information can interpolate the stimuli correctly, while mechanisms relying only on binocular motion detection will perceive ambivalently oriented arrowheads. Stimuli for the four conditions (binocular, monocular left, monocular right and dichoptic) are shown along with the mask behind which they float. (C) Motion direction and hence correct arrowhead orientation can only be derived after the binocular fusion, while monocular motion information is always ambivalent. The stimulus is the same for all four conditions, whereas the mask differs. Here, the mask and the resulting global motion path are shown for the dichoptic condition. In the monocular conditions, exclusively either the red or else the green dots were visible, whereas in the binocular case, all dots were always visible by both eyes. Results for all three experiments are given as means ±1 s.e.m.

**Figure 3:**
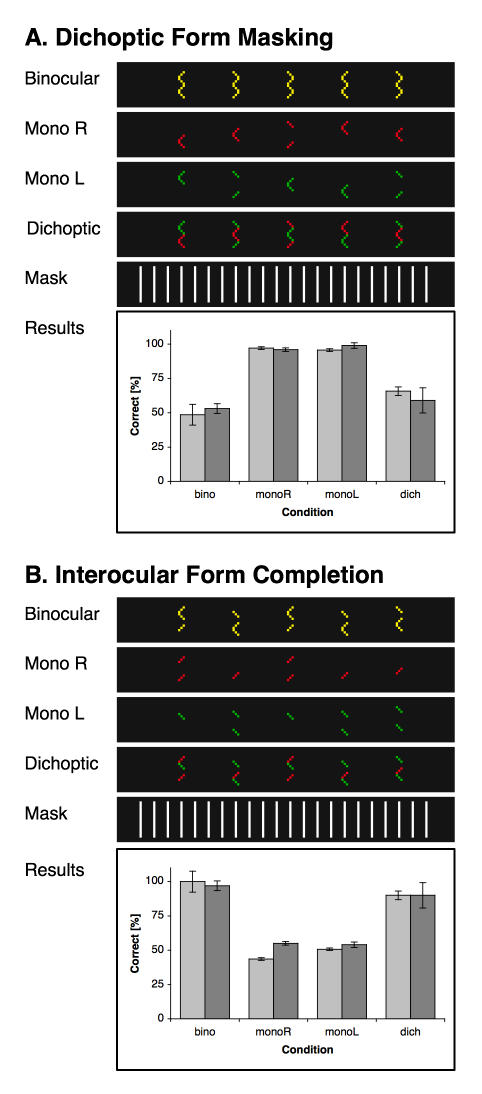
Form detection. (A) Due to dichoptic masking, arrowhead orientations may only be detected by monocular form mechanisms, while binocular ones will only perceive ambivalent cues. Stimuli are shown for the four conditions (binocular, monocular left, monocular right and dichoptic). Note that here stimuli were always shown as red dots on dark ground and that colour coding appears here only for the sake of clarity. (B) Since monocular stimuli are incomplete, detection of the arrowheads in this experiment is reserved for binocular interpolation mechanisms which have access to the interocularly combined images. Results for all experiments are shown as means ±1 s.e.m.

For monocular and dichoptic conditions, the visual input to the two eyes had to be separated. Both, red-green anaglyphs as well as LCD shutter goggles allow the presentation of both eyes' views on the same monitor. Red-green anaglyphs were used as a preferred means. As both eyes' views can be drawn on identical monitor frames, they allow a high temporal resolution (166 Hz), which is beneficial for spatio-temporal interpolation. However, in experiments 1A, 1B and 2A, colour differences between the stimuli presented to the two eyes might diminish any effects of interocular completion masking. Thus in these experiments LCD shutter goggles (CrytalEyes 3, StereoGraphics, San Rafael, CA, USA) were used, which allow the stimuli for both eyes to be maximally similar, thus preventing subjects from distinguishing between the two eyes' input simply by colour. A possible drawback would be that the images for the two eyes have to be presented on successive rather than identical frames and that thus the monocular frame rate is reduced by factor two. However, neither the reduced velocity caused by the reduced frame rate nor the resulting monocular flicker of 83 Hz had any negative impact on the subjects' perception of the interpolation stimulus (cf. the nearly perfect results for the monocular conditions in Figs. 2A, B and 3A).

### Subjects

Six subjects, between 26 and 33 of age, participated in all experiments. All were naive as to the purpose of the study and all had normal or corrected-to-normal visual acuity and normal stereopsis. Prior to the main experiment, subjects conducted a few practice trials with the standard stimulus depicted in Figure 1.

### Choice of parameters

Fahle & Poggio [3] and Morgan & Watt [12] found that inter-slit latencies between 20 and 50 ms, inter-slit distances below 20 arcmin and velocities between 1 and 10 deg/sec are optimal for spatio-temporal interpolation, while Nishida [7] used latencies of 80 ms between slits, an inter-slit distance of 32 arcmin and a velocity of 6.7 deg/sec.

In this study, we ran each experiment twice. In the small-scale version, slits were separated by 6 dots, i.e. a distance of 14.4 arcmin and a latency of 36 (red-green anaglyphs) or 72 ms (LCD shutters). In the large-scale version, the space between slits was doubled, resulting in a spatial separation of 12 dots or 28.8 arcmin and delays of 72 and 144 ms for red-green anaglyphs and LCD-shutters, respectively. Size, form and velocity of the arrowheads was kept equal in both versions.

### Presentation

Stimuli were presented on a 21-inch colour CRT monitor (Iiyama MS102DT) driven with 166 Hz by an Apple Macintosh G4 computer via an ATI Radeon 9000 graphics board. Subjects were seated in a dimly lit room and watched the stimuli from a distance of 114 cm.

Consisting of 22 slits, the stimuli subtended 5.1 and 10.2 deg in width for the small and large-scale version, respectively and 0.8 deg in height. Single dots, as they were visible through the slit masks, measured 2.4 by 2.4 arcmin.

In experiments using red-green anaglyphs, red dots for the right eye and green dots for the left eye were presented. Both dots appeared bright with a luminance of 5.0 cd/m^2^ against a dark background (0.05 cd/m^2^), resulting in a high Michelson contrast of 98%. There, stimuli were shown with 166 Hz, thus single trials (with 48 frames) lasted 288 ms and objects had a velocity of 6.6 deg/s. In binocular conditions, red and green dots were superimposed on top of each other, resulting in yellowish dots on the screen.

In experiments 1A, 1B and 2A LCD-shutter goggles were used, which caused the effective monocular refresh rate to be reduced to 83 Hz. As a result, the 48 frames lasted 576 ms and the object velocity was reduced to 3.3 deg/s. In these experiments dots were uniformly red (5.0 cd/m^2^). However, note that for the sake of easy comparability, in the figures stimuli are always depicted as red-green anaglyphs, even if they were actually presented in the experiments using LCD shutter goggles.

## Results

Two experiments addressed the motion and the form aspect of the spatio-temporal interpolation mechanism individually. In the first experiment, in which motion detection was tested, the form cue was unambiguous, whereas in the second experiment, which tested the form information used, motion direction was unequivocal.

As mentioned above, all experiments followed the same logic, in that four conditions were tested. The dichoptic condition represents the critical test for the mechanism in question. In experiments 1A, 1B and 2A, stimuli were *dichoptically masked* and thus presented only monocular detectors with reliable information. In contrast, in experiments 1B and 2B, we presented stimuli that are *interocularly completing* each other, thereby providing only binocular mechanisms with sufficient cues.

Next to the critical dichoptic condition, we conducted monocular and binocular controls. In the monocular controls, only the left or right-eye image of the dichoptic stimulus was shown to the left or right eye, respectively; whereas in the binocular control, the full stimulus of the dichoptic condition was presented to both eyes alike.

### Experiment 1A: Dichoptic motion masking I

Experiments 1A and 1B tested whether monocular detectors are involved in spatio-temporal interpolation by using dichoptically masked motion stimuli. While in experiment 1B monocular motion directions opposed each other and prevented thereby an unambiguous binocular motion percept, experiment 1A used spatial summation in order to mask motion direction.

In the two monocular control conditions of experiment 1A (‘monoR’ and ‘monoL’), the standard stimulus depicted in the Methods section was projected either to only the left or the right eye, while the partner eye was presented with a dark screen. Arrowheads were separated by four times the inter-slit distance and the global motion direction hence could be derived unambiguously. In fact, subjects performed nearly perfectly (cf. Results in Fig. 2A). For the critical dichoptic condition, two sets of standard stimuli were presented dichoptically, i.e. one to the left, the other to the right eye. The two sets were presented with an offset of two slits (that is a phase difference of 180 deg) to each other, so that in the combined stimulus arrowheads were separated by two times the inter-slit distance. The rationale is that for putative monocular mechanisms the motion direction is well defined, whereas for binocular mechanisms that collapse monocular views before computing motion direction on basis of these collapsed images, motion direction is ambiguous as arrowheads appear alternately in even and odd-numbered slits and thus merely seem to wobble back and forth. This latter point was controlled in the binocular condition by presenting arrowheads with twice the inter-slit distance. Subjects performed on chance level (cf. Results in Fig. 2A).

In the critical dichoptic condition and with the large-scale version, subjects did not perform better than chance (40.0%±9.21). They reported having had no stable percept of the arrowheads at all indicating that the interpolation process has no access to monocular motion information.

Unfortunately, this experiment could not be conducted in the small-scale version. The distance between the arrowheads in the left and right-eye views was so small that, rather than superimposing the stimuli for the two eyes, subjects fused them into 3D views, thereby annihilating the masking effect. Thus a second experiment had to be conducted in order to test the issue for the small scale.

### Experiment 1B: Dichoptic motion masking II

In experiment 1B we presented two sets of arrowheads moving in opposite directions. Here, binocular mechanisms were prevented from the interpolation process as they could merely read out ambiguous motion information.

In the monocular control conditions (‘monoL’ and ‘monoR’), subjects saw the standard stimuli that were described in the Methods section (cf. Fig. 1) and had to indicate the orientation of the arrowheads as described above. As expected, they performed nearly perfectly (cf. Fig. 2B).

To build up the stimuli for the binocular condition, two sets of arrowheads were used. In each, arrowheads had the same orientation but were moving in opposite directions (one to the left, the other to the right). The two sets were superimposed and presented alike to both eyes. As a result, the stimuli contained no reliable motion information and, accordingly, subjects had no stable percept of the arrowheads and performed at chance level.

In the critical dichoptic condition (see the example shown in Fig. 2B), the two sets of arrowheads were presented dichoptically rather than binocularly, that is each eye was presented with one of the sets. The rational is, that if interpolation were computed by monocular mechanisms, then each of them would detect their motion direction and interpolate their form information into their global form. As a result, subjects would perceive the veridical stimulus, namely two sets of identically oriented arrowheads moving into opposite directions. In contrast, if only binocular motion detection were involved, motion information would be equivocal (much like in the binocular condition) and subjects would either see no interpolated stimuli at all or follow any spuriously dominant motion direction and perceive two arrowheads pointing in opposite directions. Neither case would subserve them with reliable information.

In the actual dichoptic test, subjects did not perform better than chance (60.0%±3.1 and 52.0%±5.5 for the small and large-scale version, respectively). They reported perceiving either arrowheads with different orientations in the same display or no interpolated stimulus at all. The results show that the spatio-temporal interpolation mechanism fails to perceive the stimulus in the dichoptic condition and hence demonstrate that the interpolation mechanism has no access to the reliable monocular information but rather has to rely entirely on binocular motion information.

### Experiment 1C: Interocular completion

Using interocular motion stimuli, experiment 1C tested the alternative hypothesis, namely that spatio-temporal interpolation can rely on purely binocular motion information.

In the binocular control condition, the standard stimulus was presented to the two eyes. As expected, subjects perceived the arrowheads veridically and performed nearly perfectly (cf. Fig. 2C). For the critical dichoptic condition, alternate slit crossings were directed solely to the left and right eyes of the subjects. Since each successive slit crossing in the standard stimulus had a spatial phase shift of 90 degrees, in the dichoptic condition phase shifts within each eye were separated by 180 degrees, making monocular motion information ambiguous. This latter point was confirmed by the results in the monocular conditions, in which subjects saw either only the slit crossings directed to the left or else right eye, and performed at chance level accordingly.

When subjects were confronted with the dichoptic stimuli they performed nearly perfectly (87.9%±3.9 and 93.0%±3.1 for the small and large-scale version, respectively) and reported a stable percept of the arrowheads.

Taken together, while experiments 1A and 1B show that monocular motion information does not contribute to spatio-temporal interpolation, experiment 1C demonstrates that the interpolation mechanisms exclusively rely on binocular motion signals instead.

### Experiment 2A: Dichoptic form masking

Using dichoptic form-masking stimuli, experiment 2A investigated whether the spatio-temporal interpolation mechanism can process monocular form information.

Stimuli for the dichoptic condition were derived from the standard stimulus in that each single arrowhead was replaced by a pair of two arrowheads, one for each eye, presented in one of four spatial formations. The left-eye arrowhead was either presented vertically (1) *on top of* or (2) *below* the right-eye arrowhead. Alternatively, (3) the left-view arrowhead was presented centrally and the right-view arrowhead was split up into two strokes, one positioned on top of, the other below the left-view arrowhead, (4) or vice-versa (cf. the examples in Fig. 3A). Each of the four formations thus showed a closed-line figure, and these figures could be oriented either way within the same stimulus and per-se gave no clue as to the orientation of the arrowheads (cf. the binocular condition in Fig. 3A). As in the first experiment, subjects had to indicate the orientation of the arrowheads.

As controls, in the binocular condition all arrowheads and strokes were presented binocularly to the two eyes. Subjects could not identify individual arrowheads and performed at chance level. In left and right monocular control conditions, only the left or right-eye view was shown to the left and right eye, respectively, while the partner eye was presented with a blank screen. In both monocular conditions, subjects performed nearly perfectly.

In the critical dichoptic task two reliable monocular views were presented separately to the two eyes which mask each other. If the spatio-temporal interpolation mechanism can read out monocular form information then the result should be two perfectly identified sets of arrowheads. However, since the two monocular views mask each other when merged into one binocular stimulus, binocular interpolation mechanisms cannot succeed here.

Subjects identified arrowhead orientations at chance level (65.7%±5.5 and 59.1%±4,9 for the small and large-scale version, respectively). They reported having had a clear percept of sigma-shaped figures rather than of individual arrowheads in most cases. However, some subjects reported that in some cases the upper and lower arrowhead did not appear fully aligned and could hence be separated. This artefact results from the means of presentation. In order to separate the views for the left and the right views with LCD shutter goggles, monocular views have to be presented on successive frames, that is with a temporal offset of one monitor refresh (6.0 ms). Since the interpolation mechanism transforms temporal delays into spatial offsets, the two arrowheads for the two eyes appear with an offset of half a dot width to each other. A second difficulty occurs when subjects stop focussing their eyes on the same point in depth. When they instead look at points farther or nearer than the stimulus depth layer then interpolated arrowheads might appear with an artificial disparity, i.e. with an artificial separation cue. However, these cases were rather seldom reported but may account for the quite good performance of nearly 66% in the dichoptic condition. In any case, subjects could not use these artefacts consistently, so that their overall performance is well below the threshold of 75%. Hence it can be assumed that the interpolation mechanism has no access to monocular form information.

### Experiment 2B: Interocular form

In order to examine whether spatio-temporal interpolation instead relies on binocular form information, experiment 2B introduced a dichoptic form stimulus (Fig. 3B). Arrowheads in this task can only be detected by mechanisms with access to binocularly fused images.

Stimuli in this task consisted of three diagonal strokes of five dots length each, arranged vertically on top of each other. The upper and the lower stroke had the same randomized orientation and a vertical distance of seven dots between them. The middle stroke had always the opposite orientation and was aligned with either the upper or the lower one, thereby forming an arrowhead of the desired orientation.

In the binocular controls, all strokes were shown to both eyes, thus subjects here always saw an arrowhead with a flanking stroke either on top or below the former. In the monocular left and right controls the eye in question saw in each target position randomly either the two outer or else the middle stroke, while the partner eye was presented with a blank screen; i.e. in these control tests no full arrowhead was on display. As expected, subjects performed nearly perfect in the binocular and at chance level in the monocular condition.

For the dichoptic test, the two monocular views were superimposed with the result that binocular but not monocular interpolation mechanisms were enabled to perceive all the necessary form information to master the task.

In the critical dichoptic condition subjects identified the targets to high degree (90.0%±1.5 and 90.0±4.18 for the small and the large-scale version, respectively), clearly implying the involvement of binocular form detection in the spatio-temporal interpolation process.

## Discussion

Spatio-temporal interpolation describes the subjective visual illusion that a stimulus is continuously presented in full when in fact it is merely shown moving behind a slit mask so that only slit-wide impressions of it can be seen at any given point in time. For the observer to be able to perceive the whole Gestalt, form and motion detection must act jointly. While the various slit images need to be received and stored, the stimuli's global motion direction determines the correct order in which the slit-views have to be reassembled into a whole form again.

### Binocular motion and form detection subserves spatio-temporal interpolation

The first experiment showed that only binocular but not monocular motion information can be used in spatio-temporal interpolation. In experiment 1A, dichoptic motion masking prevented binocular motion detectors from identifying the global motion direction and reserved the task for monocular detectors. Since subjects could not identify the resulting target orientation better than chance, we conclude that the spatio-temporal interpolation mechanisms cannot make use of monocular motion information. Experiment 1B tested the same issue with presenting monocularly well-defined but binocularly ambiguous motion information. As in the critical dichoptic condition, subjects performed at chance level, results obtained here confirm the findings in experiment 1A in that monocular motion information does not contribute to spatio-temporal interpolation. Complementarily, experiment 1C cross-checked whether binocular motion information is exploited by using a dichoptic motion signal that presented monocular detectors with ambivalent motion signals, while providing only binocular mechanisms with reliable motion information. Subjects performed with almost perfect accuracy, which indicates clearly that binocular detection is involved.

The second experiment probed the form-detection part in the interpolation process and followed the same experimental rationale. In a dichoptic form-masking task (expt. 2A), monocular form information from both eyes was reliable individually but masked each other completely so that binocular detectors were prevented from identifying the target's orientation. Subjects performed at chance level, a result supporting the notion that monocular form detection is not involved. Experiment 2B then tested whether spatio-temporal interpolation can else rely on binocular form cues. There, a dichoptically presented target element could only be identified by mechanisms with access to the binocularly fused image. Subjects identified the targets perfectly, confirming that the underlying mechanism is binocular.

Taken together we found that only binocular but not monocular motion and form detectors underlie spatio-temporal interpolation.

### Comparison between small and large-scale versions

Furthermore, the interpolation mechanism can integrate information from small and large-scale stimuli. Subjects reported that the interpolated arrowheads looked stable when the inter-slit latency was 36 and 72 ms. Only with the longest interval of 144 ms, that is when large-scale stimuli had to be observed using LCD shutter goggles, arrowheads appeared slightly deformed at their tails. However, this lesser quality did not influence psychophysical results as subjects identified the orientation of the arrowheads in the binocular in expt. 1B with similar ease as the monocular controls in expt 1C, although stimuli in 1B are presented using LCD shutters whereas those in 1C are displayed as red-green anaglyphs.

These findings relate to the results found by Fahle & Poggio [3] and Morgan & Watt [12] in that the quality of interpolation deteriorates with prolonged temporal separation between slit crossings. The fact that the thresholds obtained here are higher than the thresholds reported by Morgan & Watt may relate to the narrow dot width of the stimuli used there [12].

### Implications for interpolation models

In their articles on spatio-temporal interpolation, Burr et al. [8], [13] explained their findings with a spatio-temporal filter similar to the spatio-temporal motion-energy models forwarded independently at the same time [3], [14], [15], [16].

In their use as motion detector models, these filters are ideally activated by a stimulus drifting with the proper velocity in the proper direction. Conversely, if one knows that the detector is activated and furthermore knows the start point and the start time, then one can predict the expected time point for every position and, more importantly here, the stimulus' position for a given time. In that sense, time and place are exchangeable. Burr et al. [8], [13] argued that these models, once activated by the global motion velocity of the stimuli, might then also detect the target forms (in their case verniers) in that for them, temporal delays are interchangeable with spatial distances.

However, spatio-temporal energy models have been proven to be computationally equivalent to the monocular elaborated Reichardt detector [15], [see also 17 & 18]. Since we show here that both motion and form detection underlying spatio-temporal interpolation are *binocular*, we propose that the notion of analogy between spatio-temporal interpolation mechanisms and the *monocular* spatio-temporal motion-energy mechanisms / Reichardt detectors needs to be revised.

Furthermore our results call for a re-interpretation of some previously reported data that was taken as experimental evidence for the monocular nature of the spatio-temporal interpolation detector. Fahle & DeLuca [19] dichoptically presented two verniers of opposite orientation moving towards each other and found that subjects perceived a single vernier oriented in three-dimensional space, moving either away from the subjects or toward them. Fahle and De Luca argued that for the stereo detector to be activated properly, the verniers must have been interpolated already monocularly. However, in their displays the left and right upper stroke of the vernier appeared simultaneously behind the slits and so did the left and right lower strokes. Thus as a new explanation, we propose here that the stereo detector might have simply fused the upper and the lower monocular strokes individually into an upper and a lower three-dimensional stroke; and that then the binocular interpolation mechanism computed the real form of the stereoscopically fused strokes.

### Adjusted models of spatio-temporal interpolation

In that we question the monocular nature of the spatio-temporal interpolation mechanism, our finding reintroduces the question of what mechanisms may constitute spatio-temporal interpolation. Three candidate models seem possible:

1. A unitary spatio-temporal interpolation detector, which is binocular rather than monocular. Following Burr et al.'s [2], [8], [13] proposal that interpolation can be detected by a spatio-temporal energy mechanism, and further following the proposition [17], [20] that both, the binocular first-order and the binocular third-order, motion mechanism are also standard motion-energy detectors, one could argue that interpolation might be achieved by any of these binocular motion detectors. Since stimuli could be interpolated even for cycle frequencies of approx. 7 Hz (24 frames of 6 ms each per cycle) the employment of the third-order motion detector with its temporal cut-off frequency of 2–4 Hz, appears less likely [17], [21]. However, Burr's model remains incomplete insofar as it is “lacking a clear statement of how or by what underlying mechanism the unitary gestalt is formed” [22].
2. In the concurring retinal-painting model, binocular motion mechanisms would grasp the global motion and induce suitable smooth-pursuit eye movements. Due to the resulting retinal shift, temporally delayed dots and lines would be drawn onto the retina with a spatial displacement. While this model incorporates also a monocular stage, namely the two retinae, the actual forms are only detected on the binocular salience map. This model is subserved by the findings that area MT on the one hand plays an integrative role in initiating and controlling smooth-pursuit eye movements [e.g. 23] and on the other hand computes three-dimensional (i.e. binocular) motion [e.g. 24]. In this model motion and form can be thought of as detected by completely separate units, linked externally by the eye movements.
3. The third model incorporates two separate mechanisms for form and motion. A binocular first-order motion detector would detect the global motion direction and feed this information forward into a binocular form detector, which in turn shifts incoming slit-views accordingly and integrates across a duration of 50 to 80 ms. It might also be the binocular third-order rather than the binocular first-order motion detector that grasps the global motion and feeds this information then back into the form detector (‘saliency map’). However, as already mentioned above, the cycle frequency of 7 Hz may be too fast for the third-order motion detector [17]. Either way, this model would incorporate internal links between motion and form detectors.

### Conclusion

We have demonstrated that the mechanisms underlying spatio-temporal interpolation comprise binocular sub-mechanisms for form and motion detection. This finding contradicts earlier assumptions according to which spatio-temporal interpolation is subserved by monocular detectors, or more specific, by a monocular unitary spatio-temporal energy detector [3], [8]. From this new viewpoint, three modified models seem possible–a binocular unitary spatio-temporal motion detector, binocular motion detectors in area MT (V5) inducing smooth-pursuit eye movements which in succession turn temporal delays into spatial offsets and finally two separate binocular detectors for form and motion that are linked via feed-back loops internally.
